# Re-evaluating the Role of Partnership-Related Perceptions in Women's Preferences for Men with Masculine Face Shapes

**DOI:** 10.1177/14747049241262712

**Published:** 2024-06-17

**Authors:** Junzhi Dong, Kathlyne Leger, Anthony J. Lee, Yasaman Rafiee, Benedict C. Jones, Victor K. M. Shiramizu

**Affiliations:** 1 Department of Psychological Sciences & Health, 3527 University of Strathclyde, Glasgow, UK; 2 Division of Psychology, 7622 University of Stirling, Stirling, UK

**Keywords:** attractiveness, faces, masculinity, mate preferences, parenting

## Abstract

Many researchers have proposed that women perceive men with masculine face shapes to be less suitable as parents and long-term partners than men with feminine face shapes, causing women to find masculine men more attractive for short-term than long-term relationships. However, recent work shows that results obtained using the type of experimentally manipulated stimuli that were employed in studies presenting evidence for these claims are not necessarily observed when natural (i.e., unmanipulated) face stimuli were used to suggest that the evidence for these claims may need to be revaluated. Consequently, we tested for possible relationships between ratings of natural male faces for parenting- and relationship-related traits and shape masculinity (Study 1) and also tested whether women's preferences for shape masculinity were stronger when natural male faces were rated for short-term relationships than when natural male faces were rated for long-term relationships (Studies 2 and 3). We saw no evidence for either of these predictions, instead finding that men with more attractive faces were perceived to be better parents and better long-term partners. Thus, our findings do not support the widely held view that masculine men are more attractive for short-term relationships because they are perceived to be unlikely to invest time and effort in their romantic partners and offspring.

## Introduction

In many nonhuman species, females show strong preferences for mates displaying masculine physical characteristics (Andersson, 1994; Rosenfield et al., 2019). By contrast, there is little evidence that women show strong preferences for men displaying masculine facial characteristics (see Rhodes, 2006 and Little et al., 2011 for reviews) despite many researchers hypothesising that women will prefer mates with masculine faces because male facial masculinity is a putative cue of good genes for immunocompetence (see, e.g., Thornhill & Gangestad, 1999). The dominant explanation for the somewhat surprising finding that women do not appear to show strong preferences for masculinity in men's faces is that women perceive men with masculine face shapes to be less likely to invest resources (e.g., time and effort) in their romantic relationships and offspring than men with more feminine face shapes, causing masculine men to be less attractive as potential romantic partners than would otherwise be the case (e.g., Boothroyd et al., 2007; Jones et al., 2018; Little et al., 2002, 2011; Penton-Voak et al., 1999; Perrett et al., 1998).

Empirical evidence for this explanation comes from two patterns of results that have been widely reported in the human mate-preference literature. The first pattern of results is that women tend to perceive men with more masculine face shapes to be more likely to cheat on their romantic partners, less likely to nurture offspring, less interested in long-term relationships, and as ‘bad parents’ (e.g., Boothroyd et al., 2007; Boykin et al., 2023; Kruger, 2006; Perrett et al., 1998). The second pattern of results is that women tend to show stronger preferences for men with masculine face shapes when assessing men's attractiveness for hypothetical short-term relationships than when assessing men's attractiveness for hypothetical long-term relationships (e.g., Jones et al., 2018; Little et al., 2002; Little & Jones, 2012; Penton-Voak et al., 2003; but see also Clarkson et al., 2020; Lee et al., 2014; Stower et al., 2020). While these patterns of results have informed a great deal of research on women's mate preferences, recent work has highlighted a potentially serious limitation of the methodologies that were employed in these studies.

Studies reporting that women perceive men with masculine face shapes as unlikely to invest resources in their relationships and offspring or that women show stronger preferences for men with masculine face shapes when assessing men's attractiveness for short-term relationships than when assessing men's attractiveness for long-term relationships have typically employed stimuli in which the shape characteristics displayed by male face images were experimentally manipulated using computer graphics methods. However, many researchers have recently suggested that experimentally manipulated face stimuli may have poor ecological validity (Dong et al., 2023; Jones & Jaeger, 2019; Jones et al., 2023; Lee et al., 2021; Leger et al., 2023; Lewis, 2020, 2017; Scott et al., 2010, 2013). Indeed, several studies have recently reported that the large effects of shape characteristics on social judgments of faces that can be obtained using this method are considerably smaller (and often not significant) when natural (i.e., unmanipulated) face images were used and shape characteristics were measured from these stimuli (Dong et al., 2023; Jones & Jaeger, 2019; Lee et al., 2021; Leger et al., 2023; Scott et al., 2010, 2013).

Given the possible methodological problems with manipulated face stimuli described above, we first tested for possible relationships between women's ratings of natural (i.e., unmanipulated) male faces on a range of relationship- and parenting-related traits and a widely used index of the shape masculinity of the face images (Study 1). Using the same stimuli and methods, in Studies 2 and 3, we then investigated whether preferences for these traits and shape masculinity were modulated by the temporal context of the relationship for which women assessed men's attractiveness (short-term versus long-term). We carried out these latter two studies to test whether the effects of relationship context on women's masculinity preferences previously reported in studies using manipulated face stimuli also occur for ratings of natural (i.e., unmanipulated) face images.

## Study 1. Introduction

In Study 1, women rated natural (i.e., unmanipulated) male faces for a range of parenting- and relationship-related traits. These ratings were then subjected to Principal Component Analysis (PCA) to identify the perceptual dimensions underpinning these ratings. Finally, we tested for possible relationships between these perceptual dimensions and face-shape masculinity. Because we had no a priori predictions about the perceptual dimensions that might underpin ratings of male faces for parenting- and relationship-related traits, Study 1 was exploratory.

### Methods

#### Ethics

Procedures used in all three studies were approved by the Department of Psychological Sciences and Health (University of Strathclyde) Ethics Committee, all work was undertaken in accordance with the Declaration of Helsinki, and all participants provided informed consent.

#### Stimuli

Stimuli were 90 White male faces (mean age = 27.65 years, *SD* = 6.00 years), randomly selected from the Chicago Face Database (Ma et al., 2015). The individuals photographed posed with neutral expressions and gaze directed at the camera.

#### Ratings

Two hundred and fifty heterosexual women (mean age = 28.09 years, *SD* = 4.22 years) were randomly allocated to rate the 90 male faces for one of eight questions using a 1 (not very) to 7 (very) scale. Trial order was fully randomized. These questions were designed to reflect a wide range of parenting- and partnership-related traits. Questions were ‘How likely do you think this man is to be a good parent?’, ‘How likely do you think this man is to be a good romantic partner?’, ‘How interested do you think this man would be in having a committed, long-term relationship?’, ‘How interested do you think this man would be in having a casual, short-term relationship?’, ‘How likely do you think this man would be to cheat on their romantic partner?’, ‘How interested do you think this man is in becoming a father?’, ‘How much time and effort do you think this man would put into his romantic relationships?’, and ‘How much time and effort do you think this man would put into raising his children?’. Inter-rater agreement was high for each trait (all Cronbach's alphas ranging from 0.75 to 0.90). The mean rating for each face was calculated separately for each trait and used in analyses. Descriptive statistics for these ratings are shown in Table 1. The study was run online using Experimentum software (DeBruine et al., 2020) and participants were recruited via the Prolific participant-recruitment platform. Eligibility criteria for participation were heterosexual women between 18 and 35 years of age who had English as their first language. Participants were compensated £1.05 for taking part in the study. Identical eligibility criteria were applied to Studies 2 and 3.

**Table 1.** Descriptive Statistics for Ratings in Study 1.

Question asked when collecting ratings	Number of raters	Mean	*SD*	Cronbach's alpha
How likely do you think this man is to be a good parent?	32	3.53	1.50	0.88
How likely do you think this man is to be a good romantic partner?	31	2.39	1.42	0.89
How interested do you think this man would be in having a committed, long-term relationship?	33	3.56	1.67	0.75
How interested do you think this man would be in having a casual, short-term relationship?	32	2.84	1.81	0.75
How likely do you think this man would be to cheat on their romantic partner?	31	3.16	1.60	0.82
How interested do you think this man is in becoming a father?	30	3.21	1.66	0.90
How much time and effort do you think this man would put into his romantic relationships?	30	3.40	1.45	0.85
How much time and effort do you think this man would put into raising his children?	31	3.41	1.41	0.86

#### Measuring Face-Shape Masculinity

Face-shape masculinity was objectively assessed for each of the 90 male face images using the facefuns package (Holzleitner & DeBruine, 2021) in R (R Core Team, 2021). This method has been used to assess face-shape masculinity in many previous studies (e.g., Bartlome & Lee, 2023; Cai et al., 2019; Dong et al., 2023; Holzleitner et al., 2019; Komori et al., 2011; Leger et al., 2023). Shape components were first derived from PCA of 132 Procrustes-aligned landmark points (see Holzleitner et al., 2019 for a diagram showing these facial landmarks) on each of the 90 male faces. Masculinity scores were then calculated for each image using a vector analysis method (e.g., Bartlome & Lee, 2023; Cai et al., 2019; Dong et al., 2023; Holzleitner et al., 2019; Komori et al., 2011; Leger et al., 2023). This method uses the shape principal components (PCs) to locate each face on a female-male continuum, defined by calculating the average shape information for the 90 White female faces in the Chicago Face Database and the average shape information for the 90 male faces presented in the study. Masculinity scores were then derived by projecting each image onto this female-male vector. The 90 female faces used to calculate the average female face shape were the only 90 white female faces in the image set (i.e., not a selected subset of the available white female images) and had a mean age of 28.15 years (*SD* = 5.72 years). Higher scores indicate more masculine face shapes. No scores were more than three standard deviations from the mean (i.e., there were no extreme values, *M* = 1.00, *SD* = 0.37).

### Results

All analyses were carried out using R (R Core Team, 2021), with the kableExtra 1.3.4 (Zhu, 2021), lme4 (Bates et al., 2015), lmerTest 3.1-3 (Kuznetsova et al., 2017), jtools 2.2.3 (Long, 2022), psych 2.2.5 (Revelle, 2022), and tidyverse 1.3.1 (Wickham, 2021) packages. All data, full outputs, and analysis code are publicly available on the Open Science Framework (https://osf.io/w2xj6/).

#### PCA of Ratings

First, mean ratings for each face were subjected to PCA with oblimin rotation. This analysis revealed two PCs, explaining 54% and 28% of the variance in ratings, respectively. The two components were positively correlated (*r* = .391, *N* = 90, *p* < .001). Factor loadings of the individual traits on both PCs are shown in Table 2. We labeled the two PCs the ‘long-term oriented component’ and the ‘short-term oriented component’, respectively. Higher scores on these components indicate men perceived to be suitable for long-term relationships and as good parents and suitable for short-term relationships, respectively.

**Table 2.** Correlations Between Each Individual Trait and Scores on the Long-Term Oriented and the Short-Term Oriented Components in Study 1.

	Long-term oriented component	Short-term oriented component
How interested do you think this man would be in having a committed, long-term relationship?	**0** **.** **986**	−0.145
How likely do you think this man is to be a good parent?	**0**.**931**	0.054
How interested do you think this man is in becoming a father?	**0**.**917**	−0.056
How much time and effort do you think this man would put into raising his children?	**0**.**908**	0.068
How much time and effort do you think this man would put into his romantic relationships?	**0**.**666**	0.365
How likely do you think this man is to be a good romantic partner?	0.436	**0**.**608**
How likely do you think this man would be to cheat on their romantic partner?	0.103	**0**.**780**
How interested do you think this man would be in having a casual, short-term relationship?	−0.119	**0**.**964**

Correlations with absolute values larger than 0.5 are bolded.

#### Testing for Correlations Between Shape Masculinity and Both Long-Term Oriented and Short-Term Oriented Component Scores

Next, we used regression analyses to test for possible relationships between face-shape masculinity scores and scores on the long-term-oriented and short-term-oriented components. Face-shape masculinity scores were not significantly related to scores on either the long-term oriented component (estimate = −0.007, *SE* = 0.107, *t* = −0.068, *p* = .946) or short-term oriented component (estimate = 0.093, *SE* = 0.106, *t* = 0.875, *p* = .384). Repeating these analyses controlling for possible effects of face age showed the same pattern of results (see https://osf.io/w2xj6/ for full results of these analyses).

### Study 1. Discussion

In Study 1, PCA of ratings of men's faces for a range of parenting- and partnership-related traits produced two components. The first component, which we labeled the long-term oriented component, was strongly positively correlated with ratings for questions such as ‘How interested do you think this man would be in having a committed, long-term relationship?’ and ‘How likely do you think this man is to be a good parent?’. The second component, which we labeled the short-term oriented component, was strongly positively correlated with ratings for the questions ‘How interested do you think this man would be in having a casual, short-term relationship?’ and ‘How likely do you think this man would be to cheat on their romantic partner?’. Neither of these components was significantly correlated with men's face-shape masculinity. Thus, our results do not support the proposal that women perceive men with more feminine face shapes to be better long-term partners and/or more likely to be good parent (e.g., Boothroyd et al., 2007; Boykin et al., 2023; Kruger, 2006; Perrett et al., 1998).

## Study 2. Introduction

In Study 2, we tested whether women showed stronger preferences for masculine shape characteristics when rating the attractiveness of natural (i.e., unmanipulated) male faces for hypothetical short-term relationships than hypothetical long-term relationships. We also tested whether the relationship context for which women rated men's facial attractiveness moderated the strength of possible relationships between attractiveness and men's scores on both the long-term-oriented and short-term-oriented components.

### Methods

Study 2 used the same male face images that had been used as stimuli in Study 1. One hundred heterosexual women (mean age = 28.38 years, *SD* = 4.23 years) rated the attractiveness of the 90 male faces for either a short-term relationship or a long-term relationship using a 1 (much less attractive than average) to 7 (much more attractive than average) scale. Attractiveness ratings for short-term relationships and for long-term relationships were made in separate blocks of trials and both trial order and block order were fully randomised. Short-term and long-term relationships were defined in the same way as in many previous studies that tested for possible effects of relationship context on women's masculinity preferences (e.g., Garza & Byrd-Craven, 2023; Jones et al., 2018; Little & Jones, 2012; Penton-Voak et al., 2003).

In the short-term-attractiveness test, women were given the following information: You are looking for the type of person who would be attractive in a short-term relationship. This implies that the relationship may not last a long time. Examples of this type of relationship would include a single date accepted on the spur of the moment, an affair within a long-term relationship, and the possibility of a one-night stand.

In the long-term-attractiveness test, women were given the following information: You are looking for the type of person who would be attractive in a long-term relationship. Examples of this type of relationship would include someone you may want to move in with, someone you may consider leaving a current partner to be with, and someone you may, at some point, wish to marry (or enter into a relationship on similar grounds as marriage).

Inter-rater agreement was high for both short-term and long-term attractiveness ratings (both Cronbach's alphas > 0.96). The study was run online using Experimentum software (DeBruine et al., 2020) and participants were recruited via the Prolific participant-recruitment platform.

### Results

All analyses were carried out using R (R Core Team, 2021), with the packages kableExtra 1.3.4 (Zhu, 2021), lme4 (Bates et al., 2015), lmerTest 3.1-3 (Kuznetsova et al., 2017), jtools 2.2.3 (Long, 2022), psych 2.2.5 (Revelle, 2022), and tidyverse 1.3.1 (Wickham, 2021). All data, full outputs, and analysis code are publicly available on the Open Science Framework (https://osf.io/w2xj6/).

#### Testing for Possible Effects of Face-Shape Masculinity and Relationship Context on Attractiveness Ratings

**Figure 1.**
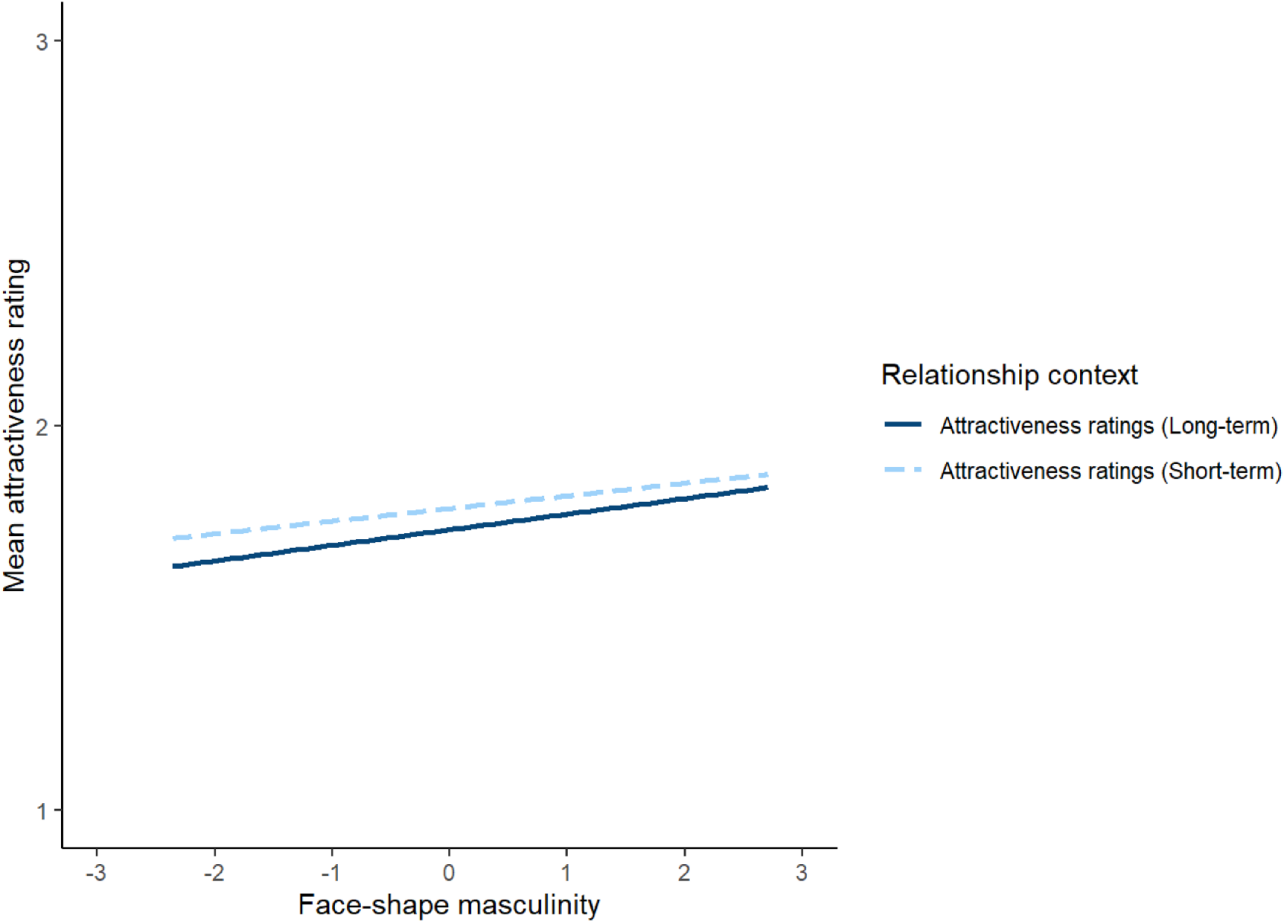
The null result for the effect of masculinity on attractiveness ratings in Study 2. In Study 2, relationship context was a within-subjects factor.

To investigate this issue, attractiveness ratings were analysed using a linear mixed effects model. The model included relationship context (effect coded so that −0.5 corresponded to short-term attractiveness and 0.5 corresponded to long-term attractiveness), face-shape masculinity scores from Study 1 (*z*-scored), and their interaction as predictors. Block order (effect coded so that −0.5 corresponded to when short-term attractiveness was rated before long-term attractiveness and 0.5 corresponded to when long-term attractiveness was rated before short-term attractiveness) was included as a covariate. The model also included by-subject random intercepts, by-stimuli random intercepts, by-subject random slopes for both the interaction between face-shape masculinity and relationship context and the main effect of block order, and by-stimuli random slopes for both relationship context and block order. This analysis showed no significant effects (see Table 3). Repeating this analysis controlling for possible effects of face age and rater age showed the same pattern of results (see https://osf.io/w2xj6/ for full results of this analysis). The null result for the effect of masculinity on attractiveness ratings are shown in Figure 1.

**Table 3.** Results of Linear Mixed Effects Model Testing for Effects of Relationship Context and Masculinity on Attractiveness Ratings (Study 2). Relationship Context was a Within-Subjects Factor in Study 2.

	Estimate	*SE*	*t*	*df*	*p*
Intercept	1.762	0.081	21.855	185.857	<.001
Relationship context	−0.052	0.035	−1.489	100	.140
Face-shape masculinity	0.026	0.054	0.475	90.089	.636
Block order (short-term first or long-term first)	−0.016	0.121	−0.136	100.177	.892
Relationship context × face-shape masculinity	0.011	0.013	0.797	17,521.425	.425

#### Testing for Possible Effects of Relationship Context, Long-Term Oriented Component Scores, and Short-Term Oriented Component Scores on Attractiveness Ratings

**Figure 2.**
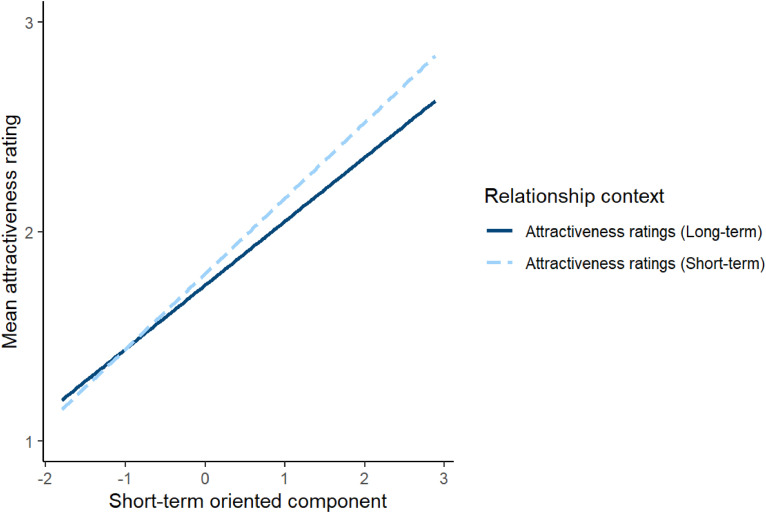
The significant interaction between relationship context and the short-term component in Study 2. In Study 2, relationship context was a within-subjects factor.

**Figure 3.**
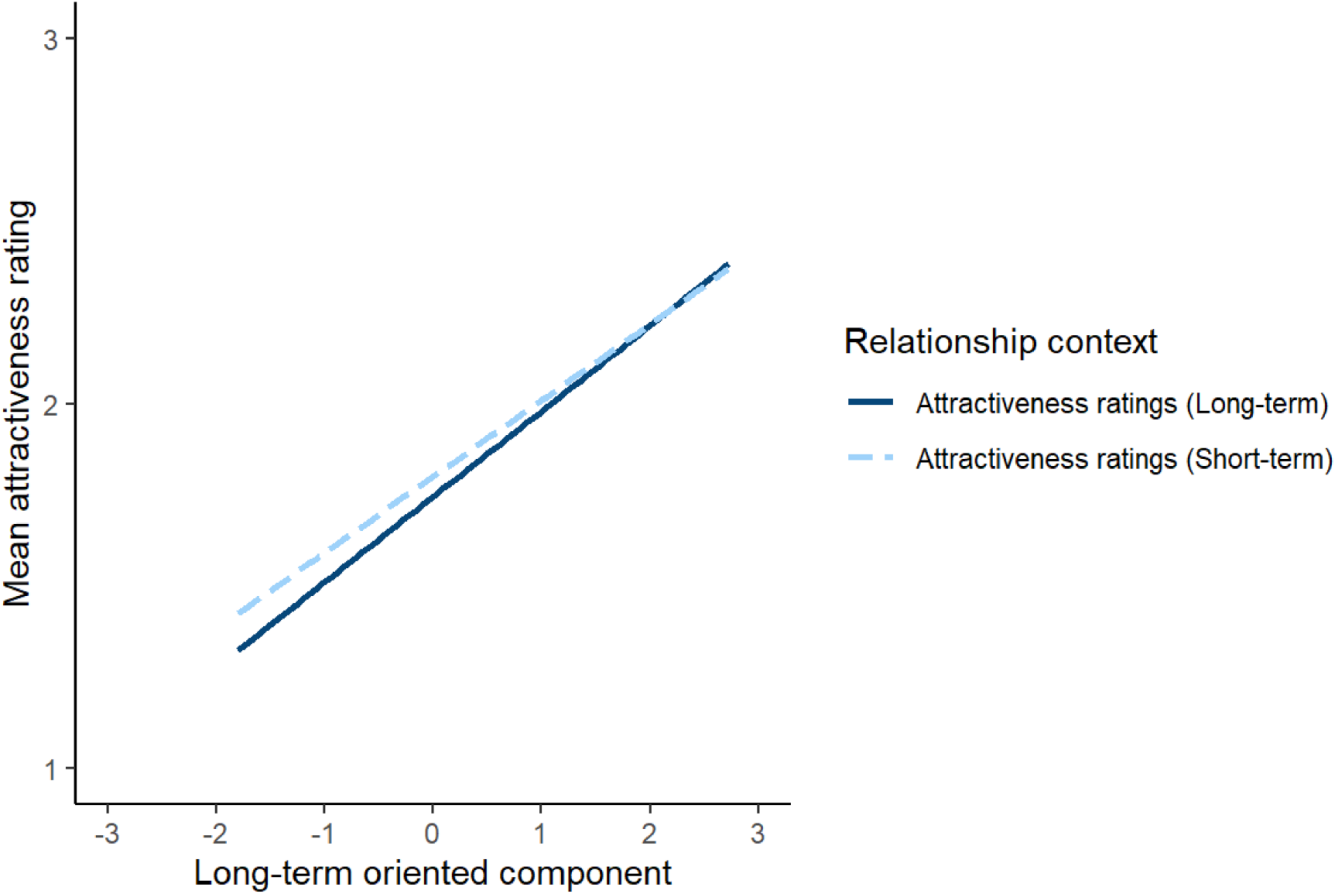
The significant interaction between relationship context and the long-term component in Study 2. In Study 2, relationship context was a within-subjects factor.

To investigate this issue, attractiveness ratings were analysed using a linear mixed effects model. The model included relationship context (effect coded so that −0.5 corresponded to short-term attractiveness and 0.5 corresponded to long-term attractiveness), the long-term oriented and short-term oriented component scores from Study 1, and the interactions between these component scores and relationship context as predictors. Block order (effect coded so that −0.5 corresponded to when short-term attractiveness was rated before long-term attractiveness and 0.5 corresponded to when long-term attractiveness was rated before short-term attractiveness) was included as a covariate. The model also included by-subject random intercepts, by-stimuli random intercepts, by-subject random slopes for the interactions between component scores and relationship context and the main effect of block order, and by-stimuli random slopes for both relationship context and block order. The results of this analysis are shown in Table 4. The significant main effects of the long-term-oriented and short-term-oriented components indicate that women generally considered men who scored higher on these components to be more attractive. However, the positive estimate for the significant interaction between relationship context and long-term oriented component scores indicates that scores on the long-term oriented component were more positively correlated with ratings of men's attractiveness for long-term than short-term relationships. Similarly, the negative estimate for the significant interaction between relationship context and short-term oriented component scores indicates that scores on the short-term oriented component were more positively correlated with ratings of men's attractiveness for short-term than long-term relationships. The significant interactions between relationship context and the short-term and long-term oriented components are shown in Figures 2 and 3, respectively. Repeating this analysis controlling for possible effects of face age and rater age showed the same pattern of results, except that the interaction between relationship context and the long-term oriented component score that was significant in our initial analyses (*p* = .034) was no longer significant when we control for the effects of rater age and face age (*p* = .081). Full results of this analysis are given at https://osf.io/w2xj6/.

**Table 4.** Results of Linear Mixed Effects Model Testing for Effects of Relationship Context and Both Long-Term Oriented and Short-Term Oriented Component Scores on Attractiveness Ratings (Study 2). Relationship Context was a Within-Subjects Factor in Study 2.

	Estimate	*SE*	*t*	*df*	*p*
Intercept	1.774	0.062	28.490	117.55	<.001
Relationship context	−0.052	0.035	−1.479	102.225	.142
Long-term oriented component score	0.152	0.028	5.382	152.465	<.001
Short-term oriented component score	0.391	0.032	12.141	172.407	<.001
Block order (short-term first or long-term first)	−0.035	0.093	−0.373	89.517	.710
Relationship context × Long-term oriented component score	0.032	0.015	2.133	314.083	.034
Relationship context × Short-term oriented component score	−0.059	0.019	−3.095	111.025	.002

### Study 2. Discussion

Analyses of attractiveness ratings in Study 2 revealed no evidence that the relationship context for which men's attractiveness was rated moderated the strength of women's preferences for male faces with masculine shape characteristics. However, women showed stronger preferences for male faces that scored high on the long-term oriented component when assessing men's attractiveness for long-term relationships than when assessing men's attractiveness for short-term relationships and showed stronger preferences for male faces that scored high on the short-term partner oriented when assessing men's attractiveness for short-term relationships than when assessing men's attractiveness for long-term relationships. Collectively, these results do not support the proposal that women show stronger preferences for men with masculine face shapes for short-term relationships (e.g., Jones et al., 2018; Little et al., 2002; Little & Jones, 2012; Penton-Voak et al., 2003). However, Study 2's results do suggest that the effects of perceptions of men's parenting ability and interest in committed long-term relationships on women's mate preferences may be moderated by the temporal context of the relationship for which men's attractiveness is assessed.

## Study 3. Introduction

Relationship context was a within-subjects factor in Study 2 (i.e., the same women assessed men's attractiveness for both short-term and long-term relationships, with attractiveness for short-term and long-term relationships being assessed in separate blocks of trials). Bressan (2021) recently presented results of reanalyses of open-access data sets that suggested previously reported effects of relationship context on face preferences may be an artefact of order and/or carry-over effects that can occur when relationship context is a within-subjects factor in the study design. Consequently, we repeated Study 2, this time using a study design in which women were randomly allocated to rate men's attractiveness for *either* a short-term relationship *or* a long-term relationship (i.e., when relationship context was a between-subjects factor).

### Methods and Results

All aspects of the methods used in Study 3 were identical to those used in Study 2, except that, in Study 3, 160 heterosexual women (mean age = 28.04 years, *SD* = 4.25 years) were randomly allocated to rate the attractiveness of the male faces for either a short-term relationship or a long-term relationship (i.e., relationship context was a between-subjects factor). Inter-rater agreement was high for both short-term and long-term attractiveness ratings (both Cronbach's alphas > 0.96). All data, full outputs, and analysis code are publicly available on the Open Science Framework (https://osf.io/w2xj6/).

#### Testing for Possible Effects of Face-Shape Masculinity and Relationship Context on Attractiveness Ratings

**Figure 4.**
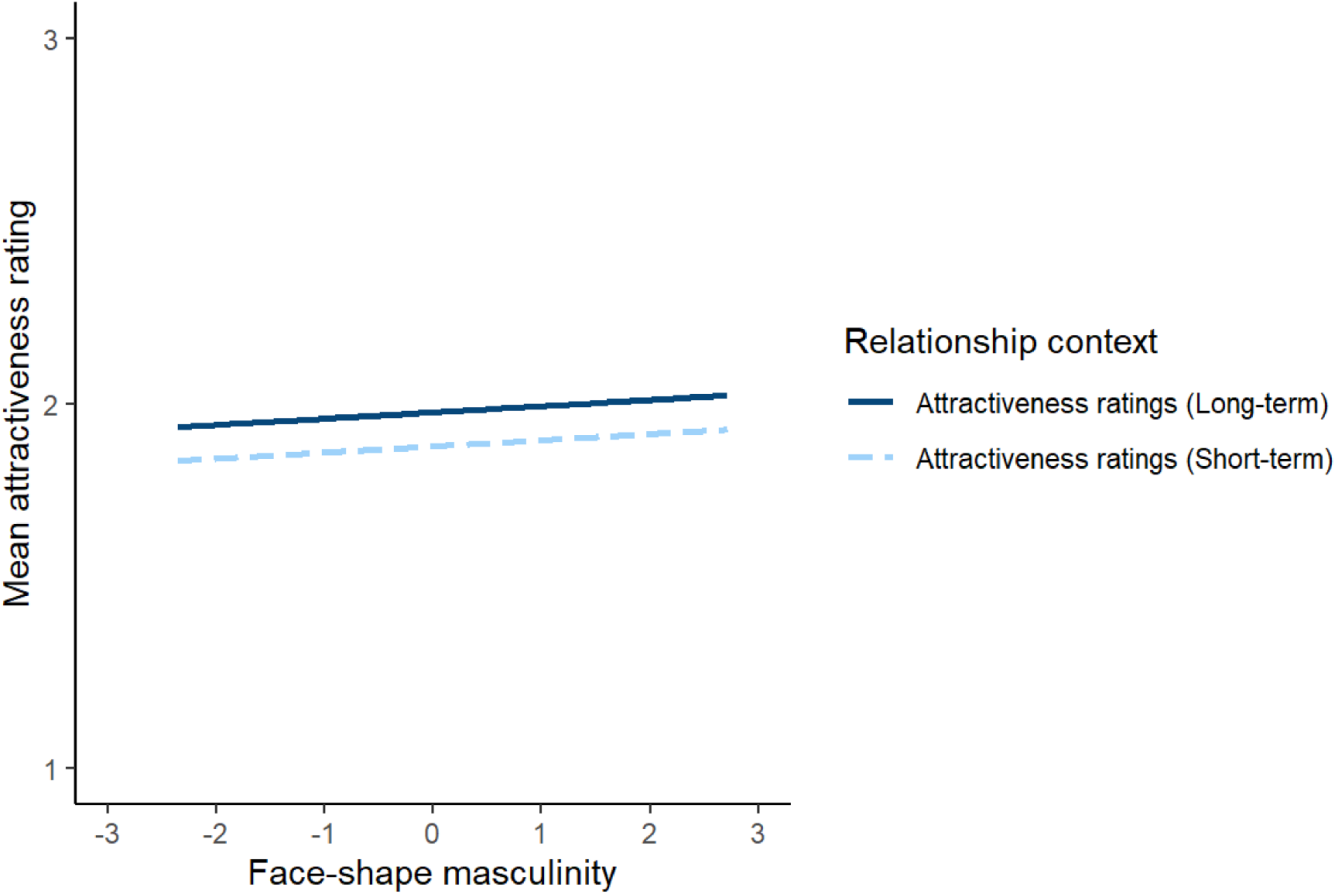
The null result for the effect of masculinity on attractiveness ratings in Study 3. In Study 3, relationship context was a between-subjects factor.

To investigate this issue, attractiveness ratings were analysed using a linear mixed effects model. The model included relationship context (effect coded so that −0.5 corresponded to short-term attractiveness and 0.5 corresponded to long-term attractiveness), face-shape masculinity scores from Study 1 (*z*-scored), and the interaction between relationship context and face-shape masculinity as predictors. The model also included by-subject random intercepts, by-subject random slopes for face-shape masculinity, by-stimuli random intercepts, and by-stimuli random slopes for relationship context. This analysis showed no significant effects (see Table 5). In particular, the non-significant interaction between relationship context and face-shape masculinity indicates that relationship context did not significantly moderate masculinity preferences. Repeating this analysis controlling for possible effects of face age and rater age showed the same pattern of results (see https://osf.io/w2xj6/for full results of this analysis). The null result for the effect of masculinity on attractiveness ratings is shown in Figure 4.

**Table 5.** Results of Linear Mixed Effects Model Testing for Effects of Relationship Context and Masculinity on Attractiveness Ratings (Study 3). Relationship Context was a Between-Subjects Factor in Study 3.

	Estimate	*SE*	*t*	*df*	*p*
Intercept	1.93	0.077	25.058	226.589	<.001
Relationship context	0.093	0.11	0.845	160.042	.400
Face-shape masculinity	0.013	0.054	0.244	89.892	.808
Relationship context × face-shape masculinity	0.001	0.016	0.044	62.294	.965

#### Testing for Possible Effects of Relationship Context, Long-Term Oriented Component Scores, and Short-Term Oriented Component Scores on Attractiveness Ratings

**Figure 5.**
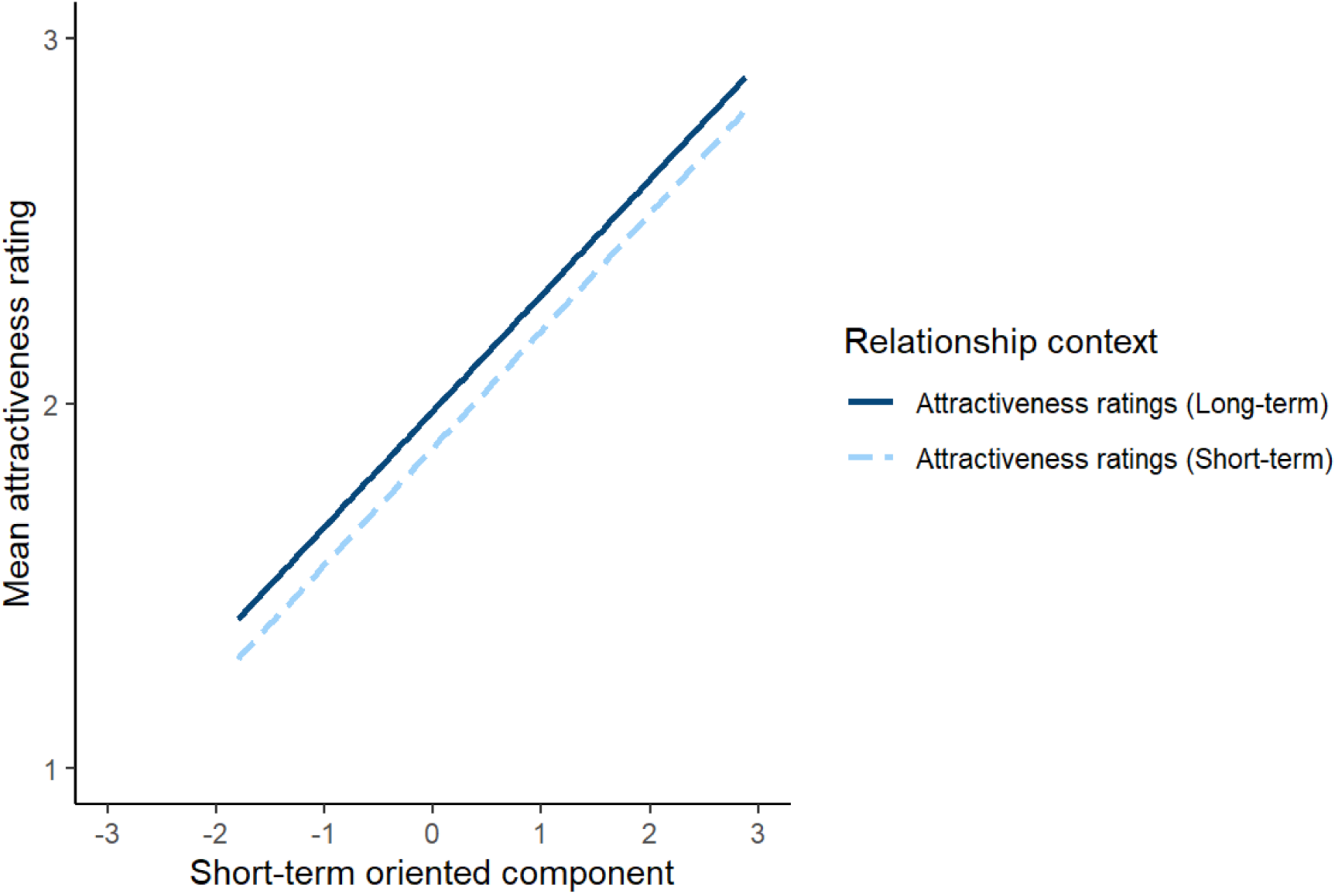
The nonsignificant interaction between relationship context and the short-term component in Study 3. In Study 3, relationship context was a between-subjects factor.

**Figure 6.**
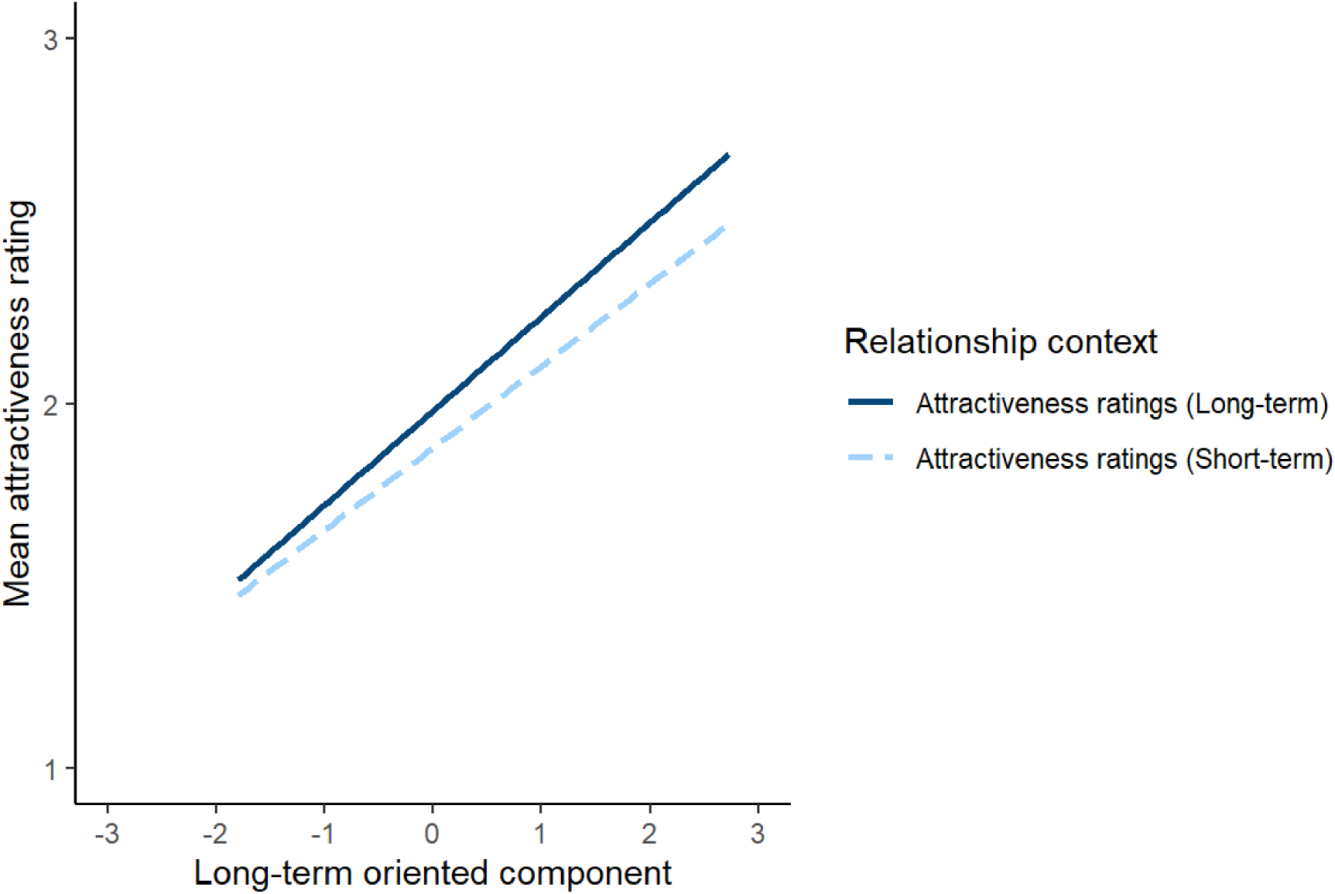
The nonsignificant interaction between relationship context and the long-term component in Study 3. In Study 3, relationship context was a between-subjects factor.

To investigate this issue, attractiveness ratings were analysed using a linear mixed effects model. The model included relationship context (effect coded so that −0.5 corresponded to short-term attractiveness and 0.5 corresponded to long-term attractiveness), the long-term oriented and short-term oriented component scores from Study 1, and the interactions between these component scores and relationship context as predictors. The model also included by-subject random intercepts, by-subject random slopes for the long-term oriented and short-term oriented component scores, by-stimuli random intercepts, and by-stimuli random slopes for relationship context. The results of this analysis are shown in Table 6. The significant main effects of the long-term-oriented and short-term-oriented components indicate that women generally considered men who scored higher on these components to be more attractive. By contrast with our results in Study 2, neither the interaction between relationship context and long-term oriented component scores nor the interaction between relationship context and short-term oriented component scores were significant. These null results are shown in Figures 5 and 6, respectively. Repeating this analysis controlling for possible effects of face age and rater age showed the same pattern of results (see https://osf.io/w2xj6/ for full results of this analysis).

**Table 6.** Results of Linear Mixed Effects Model Testing for Effects of Relationship Context and Both Long-Term Oriented and Short-Term Oriented Component Scores on Attractiveness Ratings (Study 3). Relationship Context was a Between-Subjects Factor in Study 3.

	Estimate	*SE*	*t*	*df*	*p*
Intercept	1.930	0.059	32.459	206.228	<.001
Relationship context	0.093	0.110	0.845	160.448	.399
Long-term oriented component score	0.126	0.028	4.435	122.136	<.001
Short-term oriented component score	0.402	0.031	13.093	153.85	<.001
Relationship context × Long-term oriented component score	0.039	0.028	1.391	140.05	.167
Relationship context × Short-term oriented component score	−0.010	0.037	−0.273	154.51	.785

### Study 3. Discussion

As was also the case in Study 2, analyses of attractiveness ratings in Study 3 revealed no evidence that the relationship context for which men's attractiveness was rated moderated the strength of women's preferences for male faces with masculine shape characteristics. By contrast with the results of Study 2 (in which relationship context was a within-subjects, rather than between-subjects, factor), we also found no evidence that the relationship context for which men's attractiveness was rated moderated the strength of women's preferences for male faces that scored higher on the ‘long-term oriented component’ or ‘short-term oriented component’.

## General Discussion

In Study 1, PCA of women's ratings of natural (i.e., unmanipulated) male face images for a range of parenting- and relationship-related traits revealed two components. The first component, which we labelled the ‘long-term oriented component’, reflected perceptions that a man would be interested in long-term committed relationships and likely to be a good parent. The second component, which we labelled the ‘short-term oriented component’, reflected perceptions that a man would be interested in short-term casual relationships and likely to be unfaithful. Importantly, scores on neither component were significantly correlated with men's face-shape masculinity. These null results for masculinity contrast with those of previous studies that have investigated the effects of manipulating masculinity on women's perceptions of male faces on parenting- and relationship-related traits (e.g., Boothroyd et al., 2007; Boykin et al., 2023; Kruger, 2006; Perrett et al., 1998). That we see a pattern of results for natural (i.e., unmanipulated) face images that is different to that reported by studies using face stimuli in which shape characteristics were experimentally manipulated is consistent with other recent studies finding that the large effects that manipulated face shape typically has on perceptions are considerably smaller (and often not significant) when more ecologically valid study designs and stimuli are used (Dong et al., 2023; Jones & Jaeger, 2019; Lee et al., 2021; Leger et al., 2023; Lewis, 2017, 2020; Scott et al., 2010). Indeed, other recent work using natural face images also did not observe a significant relationship between shape masculinity and women's ratings of male faces for likely parental involvement (Bartlome & Lee, 2023).

Some previous studies using face stimuli in which masculine shape characteristics were experimentally manipulated have reported that women show strong masculinity preferences when rating men's attractiveness for hypothetical short-term relationships than when rating men's attractiveness for hypothetical long-term relationships (e.g., Jones et al., 2018; Little et al., 2002; Little & Jones, 2012; Penton-Voak et al., 2003). Using the same natural (i.e., unmanipulated) stimuli we had used in Study 1, we tested whether the temporal context of the relationship for which men were being assessed moderated the possible effect of masculinity on male attractiveness. Neither Study 2 (in which relationship context was a within-subjects factor) nor Study 3 (in which relationship context was a between-subjects factor) showed evidence that women prefer masculine men more for short-term than long-term relationships. These null results are consistent with those of our Study 1 in which we found no evidence that shape masculinity was related to women's relationship- and parenting-related perceptions of men. Our null results for relationship context are also consistent with results of other recent work finding no evidence that relationship context moderated women's masculinity preferences (Clarkson et al., 2020; Lee et al., 2014; Stower et al., 2020).

Although we saw no evidence that relationship context moderated women's preferences for masculine men in Studies 2 and 3, these studies showed some evidence that relationship context moderated women's preferences for men perceived as being long-term and short-term oriented partners. In Study 2 (in which relationship context was a within-subjects factor) women showed stronger preferences for men who scored higher on the long-term oriented component when rating men's attractiveness for a long-term relationship than when rating men's attractiveness for a short-term relationship. In Study 2 women also showed stronger preferences for men who scored higher on the short-term oriented component when rating men's attractiveness for a short-term relationship than when rating men's attractiveness for a long-term relationship. However, neither of these patterns of results was observed in Study 3 (in which relationship context was a between-subjects factor). Thus, while we saw some evidence that parenting- and relationship-related perceptions have relationship-context-sensitive effects on attractiveness judgments (Study 2), these effects appear to be somewhat dependent on study design (i.e., whether relationship context was a between-subjects or within-subjects factor). Regardless of the effects of relationship context, scores on the long-term and short-term oriented components were positively related to attractiveness ratings in both studies. The positive relationships between men's facial attractiveness and scores in the long-term partner component appear to contrast with the negative relationship between attractiveness and women's ratings of male faces for likely parental involvement recently reported by Bartlome and Lee (2023).

A finding from our studies that is potentially particularly important for theories of appearance-based stereotyping and mating psychology is that parenting- and relationship-related perceptions of men's faces are underpinned by dissociable dimensions reflecting women's impressions that men are primarily interested in pursuing short-term or long-term mating strategies, respectively (see results of PCA in Study 1), rather than being underpinned by a single dimension with short-term oriented at one extreme and long-term oriented at the opposite extreme. That more attractive men are perceived as scoring higher on both of these dimensions (Study 2 and Study 3) suggests that women perceive physically attractive men as able to obtain both committed long-term partners in which they are willing to invest resources and short-term partners for more casual relationships requiring less investment of resources. Importantly, however, and by contrast with previous findings using manipulated stimuli (e.g., Boothroyd et al., 2007; Boykin et al., 2023; Kruger, 2006; Perrett et al., 1998), our results for ratings of natural (i.e., unmanipulated faces) revealed no evidence that face-shape masculinity plays any significant role in these stereotypic perceptions. Thus, our results present further evidence that findings obtained using face stimuli in which shape characteristics were experimentally manipulated do not necessarily generalise straightforwardly to ratings of natural face images that vary simultaneously on multiple dimensions (see also Dong et al., 2023; Jones & Jaeger, 2019; Lee et al., 2021; Leger et al., 2023; Scott et al., 2010, 2013).

Many researchers have suggested that women perceive men with masculine face shapes to be less likely to invest time and effort in their romantic relationships and offspring than men with more feminine face shapes and that this causes masculine men to be less attractive as potential romantic partners than would otherwise be the case (e.g., Boothroyd et al., 2007; Jones et al., 2018; Little et al., 2002, 2011; Penton-Voak et al., 1999; Perrett et al., 1998). Key pieces of evidence that are commonly cited as support for this claim are that women perceive men with more masculine face shapes to be more likely to cheat on their romantic partners, less likely to nurture offspring, less interested in long-term relationships, and as ‘bad parents’ (e.g., Boothroyd et al., 2007; Boykin et al., 2023; Kruger, 2006; Perrett et al., 1998) and that women show stronger preferences for men with masculine face shapes when assessing men's attractiveness for hypothetical short-term relationships than when assessing men's attractiveness for hypothetical long-term relationships (e.g., Jones et al., 2018; Little et al., 2002; Little & Jones, 2012; Penton-Voak et al., 2003; but see also Clarkson et al., 2020; Lee et al., 2014; Stower et al., 2020). However, these studies all used face stimuli in which shape characteristics were experimentally manipulated. By contrast with these findings, and using natural (i.e., unmanipulated) images as stimuli, we found no evidence that women's perceptions of men on relationship- and parenting-related traits are correlated with their face-shape masculinity (Study 1) or that women show stronger preferences for masculine men when rating their attractiveness for short-term relationships (Studies 1 and 2). That we observed strong positive relationships between attractiveness ratings of men's faces and women's perceptions of men on relationship- and parenting-related traits suggests that these perceptions are correlated with men's attractiveness, rather than their masculinity.
